# Indigenous skin wellness study: Exploring skin cancer awareness and UV protection practices in South Dakota Native American communities

**DOI:** 10.1016/j.jdin.2026.07.009

**Published:** 2026-08-04

**Authors:** Kianna Thelen, Meagan Kray, Leah Naasz, Jazmin Newton, Marcus L. Frohm

**Affiliations:** a Department of Dermatology, University of South Dakota Sanford School of Medicine, Sioux Falls, South Dakota; b Department of Dermatology, University of Iowa Hospitals and Clinics, Iowa City, Iowa

**Keywords:** American Indian/Alaska Native, health disparities, melanoma, preventive dermatology, skin cancer, sun protection, ultraviolet radiation

*To the Editor:* American Indian/Alaska Native (AI/AN) populations, approximately 9.7 million people, or 2.9% of the US population,^1^ bear the second-highest melanoma burden in the United States after non-Hispanic whites, with particularly elevated incidence and mortality in the Northern Plains.^2^ Yet, AI/AN individuals remain underrepresented in dermatologic research, and little is known about sun-protective behaviors, risk perception, and skin cancer knowledge in these communities. We surveyed AI/AN adults in South Dakota, home to 9 federally recognized tribes, to identify modifiable targets for culturally tailored prevention.

We conducted a cross-sectional study using a 44-item questionnaire adapted from validated skin cancer awareness instruments in consultation with tribal health educators. Items assessed demographics, ultraviolet exposure, sunscreen and tanning behaviors, skin cancer knowledge, and risk perception. Adults (≥18 years) self-identifying as AI/AN were recruited at tribal events, health fairs, and urban Native centers across South Dakota. The University of South Dakota Institutional Review Board approved the study; consent was implied by voluntary survey completion. Descriptive statistics and chi-square tests assessed associations between perceived susceptibility and sun-protective behaviors using Python (pandas).

Seventy participants completed the survey (67% female; 83% aged 31-65 years; 56% with high school/GED, 30% associate degree, 15% bachelor’s or higher). The largest groups were from Rosebud (23%) and Pine Ridge (21%). Sunscreen use was low: 24% reported year-round use, 33% used it only situationally, and 43% never used it. Just over half (54%) performed skin self-examinations, and 34% had ever used tanning beds. Participants who rated themselves as “very unlikely” to develop skin cancer were significantly less likely to use sunscreen (χ^2^ = 9.45, *P* = .009). Although 84% recognized changing moles as a warning sign, 76% incorrectly identified melanoma as the most common skin cancer, and fewer than 25% knew it is curable when detected early. Sixty percent correctly ranked non-Hispanic whites as highest melanoma risk, but nearly one-quarter mistakenly ranked AI/AN individuals as lowest risk. Most respondents (89%) reported no cultural practices tied to skin cancer prevention; suggested outreach strategies included integration into primary care visits, presence at powwows and health fairs, and culturally tailored print and social media (Table I).

**Table I:** Knowledge, behaviors, and risk perception related to skin cancer among AI/AN adults in South Dakota (*N* = 70)

Domain/item	*n* (%) or value
Female sex	47 (67%)
Age 31-65 y	58 (83%)
High school/GED as highest education	39 (56%)
Sunscreen use, year-round	17 (24%)
Sunscreen use, situational only	23 (33%)
Never uses sunscreen	30 (43%)
Performs skin self-examination	38 (54%)
Ever used a tanning bed	24 (34%)
Recognized changing moles as a warning sign	59 (84%)
Incorrectly identified melanoma as the most common skin cancer	53 (76%)
Recognized melanoma as curable if detected early	<25%
Correctly ranked non-Hispanic whites as highest melanoma risk	42 (60%)
Incorrectly ranked AI/AN as lowest melanoma risk	∼17 (24%)
Reported no cultural practices tied to skin cancer prevention	62 (89%)
Perceived susceptibility “very unlikely” × reduced sunscreen use	χ^2^ = 9.45, *P* = .009

Percentages may not sum to 100 due to rounding. Cell entries marked with a tilde (∼) are derived from reported percentages; the “<25%” entry reflects the value as originally analyzed.

*AI/AN*, American Indian/Alaska Native; *GED*, General Educational Developmental Test.

These findings reveal a clinically actionable pattern: low perceived susceptibility is associated with low sunscreen use, even as awareness of warning signs remains high. Dermatologists caring for AI/AN patients should anticipate this disconnect and explicitly address risk perception alongside warning-sign education, emphasizing that melanoma is curable when caught early.^3^ Partnerships with tribal health programs and primary care are essential where specialty access is limited, and culturally tailored messaging at community events may improve resonance.^4^ Limitations include self-reported responses, a modest convenience sample, and potential selection bias toward health-engaged participants. Larger studies across additional tribal nations are needed.^5^ AI/AN populations carry a disproportionate yet underrecognized skin cancer burden; closing this gap requires both clinical engagement and inclusion of Indigenous perspectives in dermatologic research and training.

### Declaration of generative AI and AI-assisted technologies in the writing process

Generative artificial intelligence (Anthropic Claude) was used to assist with language editing. The authors reviewed, edited, and verified all content and take full responsibility for the integrity of the work.

## Conflicts of interest

None disclosed.
